# Development of an 11-oxoetiocholanolone mini-kit for the quantification of faecal glucocorticoid metabolites in various wildlife species

**DOI:** 10.1093/conphys/coaf074

**Published:** 2025-10-24

**Authors:** Katie L Edwards, Catharine J Wheaton, Janine L Brown, Alicia M Dimovski, Kerry V Fanson, Andre Ganswindt, Stefanie B Ganswindt, Nicole Hagenah, Tamara Keeley, Erich Möstl, Bobbi O’Hara, Linda M Penfold, Samantha A Shablin, Rupert Palme

**Affiliations:** North of England Zoological Society, Chester Zoo, Science Department, Caughall Road, Chester, Cheshire, CH2 1LH, UK; Disney’s Animals, Science and Environment, Animal Programs, 1200 N. Savannah Circle E, Lake Buena Vista, FL, 32830, USA; Smithsonian Institution, Center for Species Survival, National Zoo Conservation Biology Institute, Front Royal, VA 22630, USA; La Trobe University, Department of Ecological, Plant and Animal Sciences, Melbourne, Victoria 3086, Australia; La Trobe University, Department of Ecological, Plant and Animal Sciences, Melbourne, Victoria 3086, Australia; University of Pretoria, Mammal Research Institute, Department of Zoology and Entomology, Pretoria 0002, South Africa; University of Pretoria, Mammal Research Institute, Department of Zoology and Entomology, Pretoria 0002, South Africa; University of Pretoria, Mammal Research Institute, Department of Zoology and Entomology, Pretoria 0002, South Africa; University of Queensland, School of Agriculture and Food Sustainability, Gatton, Queensland 4343, Australia; University of Veterinary Medicine, Experimental Endocrinology, Vienna 1210, Austria; Arbor Assays, Inc., 1143 Highland Dr. Suite A, Ann Arbor, MI 48108, USA; South-East Zoo Alliance for Reproduction & Conservation, 581705 White Oak Road, Yulee, FL 32097, USA; Disney’s Animals, Science and Environment, Animal Programs, 1200 N. Savannah Circle E, Lake Buena Vista, FL, 32830, USA; University of Veterinary Medicine, Experimental Endocrinology, Vienna 1210, Austria

**Keywords:** 11-Oxoetiocholanolone, corticosterone, cortisol, enzyme immunoassay, faecal glucocorticoid metabolites, kit development, non-invasive monitoring, stress, wildlife

## Abstract

As part of its mission to advance the field of wildlife endocrinology, the International Society of Wildlife Endocrinology aims to develop cost-effective antibodies and enzyme immunoassay kits that support research across a diverse range of species and sample matrices. To provide additional options for the quantification of faecal glucocorticoid metabolites (fGCMs), an antibody against 11-oxoetiocholanolone-17-carboxymethyl oxime (CMO) was generated in rabbits, and an enzyme immunoassay incorporating a horseradish peroxidase-conjugated label and 11-oxoetiocholanolone standard has been developed, designed for use with anti-rabbit IgG secondary antibody coated plates. This mini-kit was used to quantify glucocorticoid metabolites with a 5β-3α-ol-11-one structure in faecal extracts from 23 species: African and Asian elephants, Alpine chamois, American bison, Bengal tiger, blue wildebeest, blue-and-yellow macaw, brushtail possum, cape buffalo, fat-tailed dunnart, Florida manatee, ghost bat, giraffe, golden langur, Gould’s wattled bat, hippopotamus, Leadbeater’s possum, mandrill, okapi, roan antelope, samango monkey, short-beaked echidna, and western lowland gorilla. Pharmacological (adrenocorticotropic hormone challenge) and biological (inter-zoo translocation, wild capture, social disruption, illness/injury and veterinary intervention) challenges resulted in expected increases in fGCM concentrations, and in a subset of species, closely paralleled results from a previously established immunoassay against 11-oxoetiocholanolone-17-CMO. Two additional species tested, Krefft’s glider, which showed contradictory results on this assay compared to a previously validated enzyme immunoassay (EIA) and Ankole cow, where the magnitude increase post-event did not quite reach the 2-fold change criteria, highlight that differences in excreted faecal metabolites across species mean that no EIA will be suitable for all species. This assay provides a valuable new option for assessing adrenal activity across taxa using a group-specific antibody. Future studies should put similar emphasis on validation to determine optimal assay choice for measuring fGCMs in a variety of species.

## Introduction

Glucocorticoids (GCs) are widely used to evaluate adrenal responses to potentially stressful situations, although caution is warranted, as they also function as metabolic hormones, involved in diverse homeostatic processes (MacDougall-Shackleton *et al.,* 2019). Traditionally, GCs are measured in blood samples. However, blood collection itself can be a stressor that induces GC release, potentially confounding results. Moreover, blood GCs reflect a single point-in-time measure, often more indicative of acute responses, and may change quickly (Sheriff *et al.,* 2011). To overcome these limitations, particularly in field or longitudinal research, techniques have been developed to measure GCs in alternative, non-invasive matrices (Sheriff *et al.,* 2011). Faeces offer the advantage that they can be easily and repeatedly collected and avoid the need for handling of animals. They also yield more robust measures as concentrations are pooled over several hours and not subject to minor dynamic fluctuations (Palme, 2019). Consequently, over the past decades, non-invasive methods for evaluating adrenocortical activity have been increasingly adopted for wildlife studies (Ganswindt *et al.,* 2012a; Palme, 2019).

Today, GCs or their metabolites are generally measured using enzyme immunoassays (EIAs) because they are easy to use and eliminate the need for radioactivity (Palme, 2019). Circulating GCs, such as cortisol and corticosterone, are metabolized in the liver and further processed in the gut, with metabolites (fGCMs) subsequently excreted in varying proportions across species via the urine and faeces (Palme *et al.,* 2005). There are two general types of antibodies used for measuring fGCMs, referred to as parent-hormone or group-specific antibodies. Commercial cortisol and corticosterone EIAs are frequently used for measuring fGCMs, because they are readily available. However, these EIAs were designed to measure native GCs in blood or saliva, and since native GCs are mostly absent in faeces of many species, assays rely on antibodies that cross-react with related metabolites. Thus, for stress/welfare studies, best results are often obtained using group-specific EIAs that have been designed to measure specific groups of GC metabolites present in faeces (Palme and Möstl, 1997; Möstl and Palme, 2002; Möstl *et al.,* 2005). In many species, these EIAs have demonstrated a higher biological sensitivity reflected in greater increases in fGCM concentrations after acute stressful events (Fanson *et al.,* 2017; Palme, 2019). Predominant fGCMs differ across species and sometimes by sex (Touma *et al.,* 2003), so identifying optimal EIAs for assessing stress responses (and potentially differentiating between acute vs. chronic stressors) requires careful validation. This should include analytical (precision, sensitivity, specificity and accuracy) validation as well as a combination of pharmacological and biological validation (Palme, 2019). Together these ensure that data obtained are both reliable and importantly, biologically meaningful for the species and question of interest.

As part of the International Society of Wildlife Endocrinology (ISWE) mission to advance the field of wildlife endocrinology, one goal of our society is to develop antibodies and EIA kits that are cost-effective and facilitate research in a diverse range of species (Ganswindt *et al.,* 2012a). A specific need highlighted by our membership was for a greater variety of assays for the quantification of fGCMs, especially incorporating group-specific antibodies that can have increased sensitivity for non-invasive assessment of adrenocortical responses to potential stressors. To address this, we developed an antibody targeting 5β-androstane-3α-ol-11,17-dione (11-oxoetiocholanolone), coupled to bovine serum albumin (BSA) at position C-17, similar to that described by Möstl *et al.* (2002) (antibody code: UVM 72 T) and incorporated it into a mini-kit for ISWE members to be distributed by our partners at Arbor Assays, Inc. This group-specific antibody detects metabolites with a 5β-3α-ol-11-one structure, and was created using the same methodology as that previously shown to be valid for diverse species: ruminants (Huber *et al.,* 2003; Kleinsasser *et al.,* 2010; Ganswindt *et al.,* 2012b; Sid-Ahmed *et al.,* 2013; Bashaw *et al.,* 2016; Chizzola *et al.,* 2018; Zbyryt *et al.,* 2018; Özkan Gülzari *et al.,* 2019; Vogt *et al.,* 2023); elephants (Ganswindt *et al.,* 2003; Ganswindt *et al.,* 2010); zebras (Périquet *et al.,* 2017; Britnell *et al.,* 2024); birds (Kidawa *et al.,* 2014; Stocker *et al.,* 2016; De Almeida *et al.,* 2018); rodents (Franceschini *et al.,* 2007; Bauer *et al.,* 2008; Rehnus *et al.,* 2009; Chelini *et al.,* 2010; Sheriff *et al.,* 2012; Rehnus *et al.,* 2014; Majelantle *et al.,* 2023); and other species (Eguizábal *et al.,* 2013, Ganswindt *et al.,* 2014, Hulsman *et al.,* 2011, Lavin *et al.,* 2019). Here, we analysed samples from pharmacological and biological validations to explore the potential for detecting changes in adrenocortical activity for different species. Secondly, in a subset of species, we compared the performance of this new 11-oxoetiocholanolone ISWE010 EIA mini-kit to the existing assay, originally developed at the University of Veterinary Medicine, Vienna (Möstl *et al.* (2002), as well as some alternative glucocorticoid assays where a comparative approach to assay validation had been taken previously.

## Materials and Methods

### Antibody development and assay production

Antibodies were raised in rabbits against 11-oxoetiocholanolone 17-carboxymethyl oxime (CMO) linked to BSA, provided by the University of Veterinary Medicine, Vienna, and described by Möstl *et al.* (2002). Arbor Assays, Inc., developed a competitive EIA mini-kit (ISWE010) incorporating this antibody (hereafter 11-oxoetiocholanolone antibody), a directly labelled horseradish peroxidase (HRP) conjugate (also linked to 11-oxoetiocholanolone-17-CMO, hereafter 11-oxoetiocholanolone-HRP) and 11-oxoetiocholanolone (5β-androstan-3α-ol-11,17-dione) as standard. To determine working dilutions of the antibody and conjugated label, a checkerboard titration was initially conducted.

A competitive double antibody EIA was developed consisting of secondary goat-anti rabbit IgG antibody coated 96-well microtiter plates (ISWE005, Arbor Assays, Inc.), polyclonal rabbit anti-11-oxoetiocholanolone antibody, 11-oxoetiocholanolone standard (39–40 000 ng/ml) and 11-oxoetiocholanolone-HRP (ISWE010, Arbor Assays, Inc.), all stored at < −18°C until use. Assays were conducted using corresponding assay reagents (ISWE006, Arbor Assays, Inc.), with 50 μl of standard or sample added to pre-coated anti-rabbit plates, followed by 25 μl each of 11-oxoetiocholanolone-HRP label and anti-11-oxoetiocholanolone antibody before incubation for 2 h at room temperature, with shaking. Following subsequent washing to remove unbound reagents and incubation with a TMB substrate (100 μl) and halting of the reaction with 1 M HCl (50 μl), optical density was determined at 450 nm. This method was modified slightly following the beta-testing reported herein, such that the ISWE010 EIA mini-kit now supplied by Arbor Assays, Inc. (https://www.arborassays.com/product/72t-iswe-mini-kit/) is optimized for 100 μl per well of standard, control, and sample with the addition of 50 μl per well of 11-oxoetiocholanolone-HRP label and anti-11-oxoetiocholanolone antibody.

### Beta-testing

Seven laboratories were involved in the mini-kit beta testing, which involved faecal samples or their extracts from earlier studies, all stored frozen (< −18°C) to maintain fGCM stability (Palme *et al.,* 2013). This study included 72 individuals (33 male and 39 female) from 25 species. The selected set of samples for each species included periods of relatively low and relatively high levels of adrenocortical activity. For 23 individuals of 10 species, the change in circulating GCs was pharmacologically induced using an adrenocorticotropic hormone (ACTH) challenge (see Table 1 for methodological details). For 51 individuals representing 18 species, the stimulus was a biological challenge predicted to be associated with a change in circulating GCs (e.g. transport, medical exam; details in Table 2). Extraction procedures for each species followed established species-specific protocols within each lab. Protocols varied by sample pre-processing—wet vs. dry (lyophilized) extraction, the type (ethanol or methanol) and concentration (60–90%) of solvent, and the absence (NC) or inclusion of a concentration (3 or 5-fold) step. Faecal extracts were diluted in assay buffer (ISWE006, Arbor Assays, Inc.) and evaluated by tests for parallelism between faecal dilutions and the 11-oxoetiocholanolone standard curve (Supplementary Material, Fig. S1), and in response to ACTH or biological challenges.

**Table 1:** Median baseline fGCM concentration (ng/g) and peak response (fold-change) to ACTH challenges in 10 species measured with the newly developed 11-oxoetiocholanolone enzyme immunoassay (ISWE010 mini-kit). Species with fold-change increases in fGCMs ≥2 were considered validated

**Species**	**Sex (N)**	**Extraction** ^**a**^				**ACTH details**	**Ethical approval**	**Sampling frequency used for validations**	**Baseline vs. event**		**Baseline concentration (ng/g)**	**Peak response (fold-change)**	**Time to peak**
American bison, *Bison bison*	Male (1)	D	e	90	NC	Intramuscular injection of 1200 IU (1.5 IU/kg) synthetic ACTH (ACTHAR gel; Mallinckrodt Pharmaceuticals, USA)	This study was approved by SEZARC Internal Animal Care and Use Committee, reference PR-2018-01	Daily samples 6 days pre- and 11 days post-ACTH challenge.	Median pre	Peak post	2.1	14.6	36 h
Blue wildebeest, *Connochaetes taurinus* ^b^	Female (1)	D	e	80	NC	Intramuscular injection of 400 IU (1–2 IU/kg) of synthetic ACTH (Synacthen Depot©, Novartis South Africa (Pty) Ltd, Johannesburg)	This study was conducted with the approval of the University of Pretoria Animal Ethics committee (Reference V055–14)	Three samples collected within 24 h pre- compared to 3 samples within 24-h post-ACTH challenge	Median pre	Peak post	2022.5	2.7	23 h
	Male (1)	D	e	80	NC						881.5	12.1	23 h
Blue-and-yellow macaw, *Ara ararauna* ^c^	Female (4)	W	m	60	NC	Intramuscular injection of 0.5 mg/kg of synthetic ACTH (Synacthen® Depot, Novartis Pharma, United Kingdom)	Relevant authorizations to carry out the research were obtained from the Biodiversity Authorization and Information System (SISBIO/protocol No. 44745–1), and from the Animals Use Ethic Committee (CEUA, protocol # 52/2014) from Palotina Sector of the Federal University of Paraná	Samples collected 2 h pre- and up to 48 h post-ACTH challenge; matched samples were collected 15 days later to act as a baseline/control	Median control	Peak post	4.2–13.8	16.6–70.3	8–24 h
Brushtail possum, *Trichosurus vulpecula* ^d^	Female (3)	D	m	80	NC	Intramuscular injection of 1 ml of synthetic ACTH (Synacthen, Novartis Australia)	The use of animals in this project was approved by The University of Sydney (project number: 2018/1305), Macquarie University (AEC Ref. No.: 2016/023–6), and National Parks and Wildlife Services NSW (Permit no. SL100443 and SL101568)	Daily samples for 9 days (pre on day of capture and ACTH given)	Median pre	Peak post	177.1–1241.3	3.9–15.0	2–3 d
	Male (1)	D	m	80	NC						560.2	2.3	3 d
Cape buffalo, *Syncerus caffer* ^e^	Female (1)	D	e	80	NC	Intramuscular injection of 150 IU of synthetic ACTH (Synacthen Depot©, Hoffman La Roche AG)	The study was performed with approval of the Ethics and Scientific Committee of the National Zoological Gardens of South Africa, Pretoria (Reference # P10/33)	Three samples collected up to 18 h pre- and three samples 13–27 h post-ACTH challenge	Median pre	Peak post	1791.9	10.8	27 h
	Male (1)	D	e	80	NC			Samples collected 9 h pre- and 4–25 h post-ACTH challenge			2953.9	11.5	22 h
Giraffe, *Giraffa camelopardalis*^f^	Male (1)	D	e	80	NC	Intramuscular injection with Synacthen Depot (Novartis) at an estimated dose 1 IU/kg	The study of Giraffe 1 complied with relevant ethical guidelines in South Africa and was conducted with permission of the Animal Use and Care Committee (#EC074–12) of the University of Pretoria, South Africa and with permission of the NZG, which owned the animal	Two samples pre- and four samples up to 30 h post- ACTH challenge	Median pre	Peak post	543.4	24.8	30 h
Golden langur, *Trachypithecus geei*^g^	Female (1)	D	e	80	NC	Intramuscular injection with 6 IU/kg body weight ACTH (corticotropin carboxymethylcellulose injection; Ferring Pharmaceuticals, Ahmedabad, India)	The research complied with protocols approved by the animal ethical committee of Bodoland University and adhered to the legal requirements of India	Daily samples 1 day pre- and 5 days post-ACTH challenge	Median pre	Peak post	757.7	2.9	48 h
Roan antelope, *Hippotragus equinus*^h^	Female (1)	D	e	80	NC	Intramuscular injection (1 IU/kg) with Synacthen depot (Novartis, South Africa).	The study was performed with the approval of the University of Pretoria Animal Use and Care Committee (Reference V072–17)	Three samples collected pre-ACTH injection (day of and two days prior), followed by three samples within 24 h post-injection	Median pre	Peak post	1107.7	8.7	13.3 h
	Male (1)	D	e	80	NC						722.5	13.2	15.5 h
Samango monkey, *Cercopithecus albogularis*^i^	Female (1)	D	e	80	NC	10 IU (1.1 IU/kg—1.5 IU/kg) synthetic ACTH (Synacthen®, Novartis, Australia) intramuscularly.	The study was performed with the approval of the National Zoological Gardens Ethics Committee (Reference P10/27)	Three samples collected over the 4 days prior to ACTH injection, and three samples collected 18–84 h following.	Median pre	Peak post	1850.2	6.8	67 h
	Male (1)	D	e	80	NC						1686.4	4.6	18 h
Short-beaked echidna, *Tachyglossus aculeatus*^j^	Female (2)	D	m	80	C3	Injected with 1 ml Synacthen (250 μg Tetracosactrin; Mallinckrodt Pharmaceuticals) while under general anaesthesia	This project was approved by the University of Queensland’s Office of Research Ethics NEWMA committee	Daily samples collected 5 days pre- and 10 days post injection.	Median pre	Peak post	19.6–76.4	1.9–4.1	5–6 d
	Male (2)	D	m	80	C3						9.0–37.1	2.2–2.7	1–3 d

a ^a^Extractions were conducted using either wet (W) or dried (D) faeces, 60–90% methanol (m) or ethanol (e) and were either concentrated three-fold (C3) or not (NC).

b ^b^ Wolf *et al.* (2021)

c ^c^ De Almeida *et al.* (2018)

d ^d^ Cope *et al.* (2022)

e ^e^ Ganswindt *et al.* (2012b)

f ^f^ Bashaw *et al.* (2016)

g ^g^ Sarmah *et al.* (2017)

h ^h^ Kamgang *et al.* (2022)

i ^i^ Scheun *et al.* (2020)

j ^j^ Russell *et al.* (2022)

**Table 2:** Median baseline fGCM concentration (ng/g) and peak response (fold-change) to biological validation events in 17 species measured with the newly developed 11-oxoetiocholanolone enzyme immunoassay (ISWE010 mini-kit). Species with fold-change increases in fGCMs ≥2 were considered validated

**Species**	**Sex (N)** ^**a**^	**Extraction** ^**b**^				**Validation event**	**Sampling frequency used for validations**	**Baseline vs. event**		**Baseline concentration (ng/g)**	**Peak response (fold-change)**	**Time to peak** ^**c**^
African elephant, *Loxodonta africana*	Female (1)	W	m	90	C5	Translocation	Samples collected 1–2 times per week for 2 months pre- and daily for 2 months post-translocation	Median pre	Peak post	83.8	5.5	2 d
	Male (1)	D	e	80	NC	Injury—foot injury and lameness^d^	Monthly samples for 3 months injured, and 3 months not injured	Median not injured	Median injured	280.9	2.5	n/a
Alpine chamois, *Rupicapra rupicapra*	Male (1)	D	m	80	NC	Translocation^e^	Four daily samples pre- and two samples per day for 3 days post-translocation	Median pre	Peak post	1088.4	11.8	1 d
Ankole cattle, *Bos taurus ankole*	Female (1)	W	m	80	NC	Possible pregnancy loss	Samples collected 3–6 times per week for 4 weeks pre- and 3 weeks post- suspected miscarriage	Overall median	Peak	240.5	1.7	n/a
Asian elephant, *Elephas maximus*	Female (1)	W	m	90	C5	Translocation	Sample every 1–2 days for 2 weeks pre- and daily for 30 days post-translocation	Median pre	Peak post	38.8	9.6	2 d
	Male (1)	W	m	90	C5		Daily samples for 16 days pre- and 32 post-translocation	Median pre	Peak post	19.3	3.4	4 d
Bengal tiger, *Panthera tigris tigris*	Female (1)	W	m	90	NC	Translocation^f^	Five samples pre-, and one sample (first sample defecated) post-translocation	Median pre	Peak post	1229.3	4.3	3 d
	Male (1)	W	m	90	NC					1939.3	2.1	1 d
Fat-tailed dunnart, *Sminthopsis crassicaudata*	Female (2)	W	e	80	NC	Enclosure change (open field test)	7–8 samples per individual, collected opportunistically up to 6 weeks pre- and 12 weeks post-capture	Median pre	Peak post	288.2–508.5	2.5–3.7	1 d
	Male (2)	W	e	80	NC					500.8–634.2	1.9–3.9	0.4–0.6 d
Florida manatee, *Trichechus manatus latirostri*	Male (1)	W	m	80	NC	Translocation	Samples collected weekly pre- and daily for 2 weeks post-translocation	Median pre	Peak post	14.7	7.6	14 d
	Male (1)	W	m	80	NC	Surgical treatment	Samples collected 2–3 times per month pre- and three times per week for 3 weeks post-medical procedure	Median pre	Peak post	5.6	16.6	n/a
Ghost bat, *Macroderma gigas*	Mixed-sex group (1.7)	W	m	80	NC	Veterinary exam	Multiple samples collected 2 days pre- and 4 days post-event	Median pre	Peak post	536.0	28.7	1–2 d
Giraffe, *Giraffa camelopardalis*	Female (1)	W	m	80	NC	Health issues—periods of lethargy and inappetence	Samples collected ~twice per week for 20 weeks	Overall median	Median peak	440.1	2.6	n/a
Gould’s wattled bat, *Chalinolobus gouldii*	Female (3)	W	e	80	NC	Wild capture ^g^	Samples were collected at 0 h (considered as pre- due to gut transit time), and 11 h and 17 h post-capture	Pre	Peak post	99.0–169.4	1.7–12.2	11–17 h
	Male (3)	W	e	80	NC					118.4–128.0	2.7–5.1	11–17 h
Hippopotomus, *Hippopotamus amphibius*	Female (1)	W	m	80	NC	Health issues	Samples 1–3 times per week for 2 months	Overall median	Peak	16.9	10.5	n/a
Krefft’s glider, *Petaurus notatus*	Female (3)	W	e	80	NC	Wild capture ^h^	Two samples collected within 2 days of capture; five samples collected after 2 months in captivity.	Median pre	Median post	6711.9–23799.5	0.06–0.17	Pre > post
Leadbeater’s possum, *Gymnobelideus leadbeateri*	Two mixed-sex groups (1.1; 4.2)	W	e	80	NC	Wild capture	9–17 samples per group, collected opportunistically up to day 39 post-capture	Median (weeks 2–7 post)	Peak (≤1 week of capture)	73.4–324.5	3.2–10.4	2–5 d
Mandrill, *Mandrillus sphinx*	Male (1)	W	m	80	NC	Social change	Daily samples for 17 days	Overall median	Peak	71.3	4.0	n/a
Okapi, *Okapia johnstoni*	Female (1)	W	m	80	NC	Stressor + acyclicity	Daily samples for 38 days	Median pre	Peak post	621.7	4.6	n/a
	Male (1)	W	m	90	C5	Translocation	Samples collected every 1–2 days for 9 weeks pre- and 6 weeks post-translocation	Median pre	Peak post	207.1	13.1	2 d
Short-beaked echidna, *Tachyglossus aculeatus*	Female (1)	D	m	80	C3	Housing move ^i^	Samples collected following move to individual enclosures, daily for 11 days	Overall median	Peak post	19.4	7.2	2 d
	Male (1)	D	m	80	C3		Samples collected following move to individual enclosures, daily for 10 days	Overall median	Peak post	8.5	4.1	2 d
Western lowland gorilla, *Gorilla gorilla gorilla*	Male (1)	W	m	80	NC	Veterinary treatment	Samples collected 2–4 times per week for 2 months	Overall median	Peak	105.1	2.4	n/a
	Male (4)	W	m	80	NC	Social change	Eight samples per male, before, during, and after group introductions	Overall median	Peak	114.1–189.4	1.7–3.6	1 d

a ^a^In two cases, samples were collected from mixed-sex groups (designated m.f) instead of identified individuals.

b ^b^Extractions were conducted using either wet (W) or dried (D) faeces, 60–90% methanol (m) or ethanol (e) and were either concentrated three or five-fold (C3, C5) or not (NC).

c ^c^Where n/a – there was not a single event/peak but a period of increased concentrations.

d ^d^ Ganswindt *et al.* (2003)

e ^e^ Anderwald *et al.* (2021)

f ^f^ Jepsen *et al.* (2021)

g ^g^ Sandy *et al.* (2024)

h ^h^ Dimovski *et al.* (2025)

i ^i^ Russell *et al.* (2022)

### Data analyses

#### Pharmacological and biological validation

Our first aim was to determine whether the newly developed 11-oxoetiocholanolone EIA (ISWE010) could detect changes in adrenocortical activity for different species. To assess this, we examined the change in measured fGCM concentrations during periods of low and high adrenocortical activity. Unless otherwise specified in Tables 1 and 2, baseline concentrations were calculated using the median of pre-challenge concentrations, and peaks were determined as the maximum post-challenge concentration. For biological validations where the stressor was sustained (e.g. health-related stressors), the median peak was used; for stressors that were more defined (e.g. ACTH challenge, translocation), the maximal peak was used. To evaluate the magnitude of the response, we calculated the fold-increase from baseline to peak. If the peak was at least 2-fold greater than baseline, it was interpreted that the assay was able to detect biologically relevant changes in adrenocortical activity.

#### Assay comparison

Our second aim was to determine how the performance of this new 11-oxoetiocholanolone (ISWE010) EIA mini-kit compared to the existing assay, originally developed at the University of Veterinary Medicine, Vienna (Möstl *et al.* (2002). We had 21 sets of samples from 11 species that had been analysed on both assays. All profiles were visually inspected to assess similarity, baseline, peak concentrations and fold-change compared, and Pearson’s correlation was used to assess how closely correlated the results of the two assays were; correlations were run separately for each individual (Supplementary Material, Table S1). Finally, where other GC assays had been previously published for a particular sample-set, longitudinal profiles were visually assessed and the magnitude change from baseline to peak were compared with the previously determined best-preforming assay. Pearson’s correlation was used to compare data from the originally chosen vs. the newly developed assay; correlations were run separately for each individual (Supplementary Material, Table S2). Comparison assays included antibody code UVM 72a using 11-oxoetiocholanolone 3-hemisuccionate (Palme and Möstl, 1997); UVM 69a using a 5 -androstane-3,11 -diol-17-one-CMO:BSA generated antibody (Frigerio *et al.,* 2004); a cortisol mini-kit ISWE002 (Arbor Assays, Inc., Ann Arbor, MI, USA); and two corticosterone assays: antibody code CJM006 (Coralie Munro, University of California Davis, Davis, CA, USA (Watson *et al.,* 2013)) and mini-kit ISWE007 (Arbor Assays, Inc., Ann Arbor, MI, USA).

### Ethical declarations

All faecal samples were collected non-invasively and opportunistically around potentially stressful events (biological validation) or scheduled ACTH challenges (pharmacological validation). For the latter, ethical approval was obtained for each original study (Table 1).

## Results

### Assay optimization

Optimal dilutions were determined to be 1:320 000 for the new polyclonal rabbit anti-11-oxoetiocholanolone antibody, and 1:50 000 for the 11-oxoetiocholanolone-HRP conjugate. Cross-reactivities of the antibody in this mini-kit format, determined at 50% binding, were: 5β-androstan-3α-ol-11,17-dione (11-oxoetiocholanolone), 100.00%; 5β-pregnan-3α-ol-11,20-dione (alloalfaxolone), 9.62%; 5β-androstane-3,11,17-trione, 4.75%; 5β-androstane-3α,11β-diol-17-one (11β-hydroxyetiocholanolone), 0.99%; 5β-pregnane-3α,11β,21-triol-20-one (tetrahydrocorticosterone), 0.20%; 5β-pregnane-3α,11β-diol-20-one, 0.09%; cortisone, 0.06%; cortisol, corticosterone, 17β-oestradiol, progesterone and testosterone, all < 0.04%.

### Pharmacological and biological validation

The newly developed 11-oxoetiocholanolone (ISWE010) EIA mini-kit was generally successful at detecting changes in adrenocortical activity across a wide range of species. Following an ACTH challenge, fGCM concentrations increased above median baseline between 1.9 and 70.3-fold, peaking between 8 h in female blue-and-yellow macaw, *Ara ararauna*, and 5–6 days post-ACTH in female short-beaked echidna, *Tachyglossus aculeatus*, (Table 1). Following biological validation events, peak concentrations were between 1.7- and 28.7-fold higher than respective baselines (Table 2). Peaks were observed from 1 day post-translocation in the male Alpine chamois, *Rupicapra rupicapra*, and male Bengal tiger*, Panthera tigris tigris*, to up to 14 days post-translocation in the Florida manatee, *Trichechus manatus latirostri*; < 1 to 5 days following wild capture in the fat-tailed dunnart, *Sminthopsis crassicaudata*, and Leadbeater’s possum, *Gymnobelideus leadbeateri*, respectively; 1 day following a social introduction in a group of male western lowland gorillas, *Gorilla gorilla gorilla*, and 1–2 days following veterinary procedure in mixed-sex groups of ghost bats, *Macroderma gigas*.

One species, the Krefft’s glider, *Petaurus notatus*, passed analytical validation by way of parallelism and matrix interference assessment, but was considered not biologically validated. In three females, fGCMs measured on this assay were 83–94% lower immediately following wild-capture when compared to 2-months later and were poorly correlated (*r* = −0.51 to −0.61) with an alternative assay (cortisol ISWE002; Arbor Assays, Inc., Ann Arbor, MI, USA) tested in parallel, which showed the expected higher concentrations immediately post-capture (Supplementary Material Fig. S2). The biological validation for a female Ankole cow, *Bos taurus ankole*, did not reach the 2-fold increase following the potential stressor; this event was a suspected miscarriage, and concentrations showed a sustained increase, rather than a single defined peak. For species with data on both sexes, males and females showed varied concentrations, both within their baseline fGCM concentrations and the magnitude of their response; similarly, in species with multiple individuals tested, individual baselines and responses varied in concentration and magnitude.

### Assay comparison

**Figure 1:**
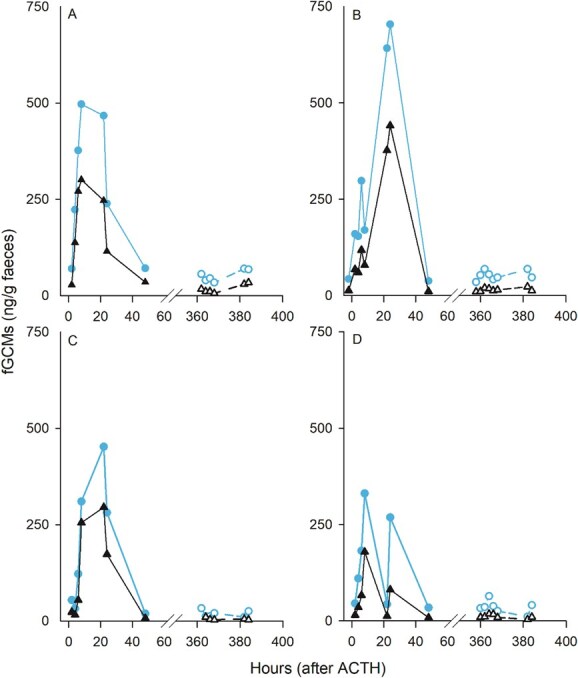
fGCM concentrations in four (**A**–**D**) female blue-and-yellow macaws (*Ara ararauna*) measured using the ISWE010 EIA mini-kit (triangle) and the previously published 11-oxoetiocholanolone EIA (circle) by Möstl *et al.* (2002). Solid lines with closed shapes represent fGCM concentrations following an ACTH challenge (time 0); dotted lines with open symbols represent baseline/control concentrations collected from the same individuals without manipulation 15-days later. Comparison data from De Almeida *et al.* (2018)

**Figure 2:**
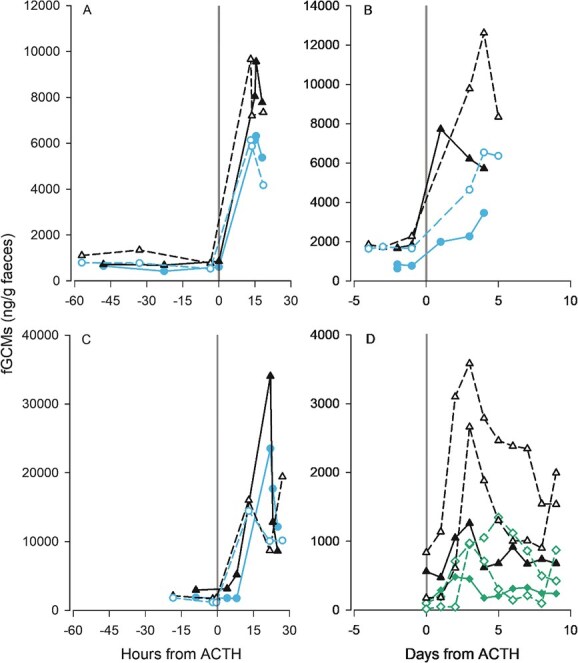
fGCM concentrations in male (solid line, closed shapes) and female (dashed line, open shapes) (**A**) roan antelope, *Hippotragus equinus*, (**B**) Samango monkey, *Cercopithecus albogularis,* (**C**) cape buffalo, *Syncerus caffer*, and (**D**) brushtail possum, *Trichosurus vulpecula* following ACTH challenge (time 0) measured using the ISWE010 EIA mini-kit (black triangle) and the previously published 11-oxoetiocholanolone EIA (circle) by Möstl *et al.* (2002) or 11-oxoetiocholanolone EIA (diamond) by Palme and Möstl (1997). Comparison data from Ganswindt *et al.* (2012b), Scheun *et al.* (2020), Cope *et al.* (2022) and Kamgang *et al.* (2022), respectively

**Figure 3:**
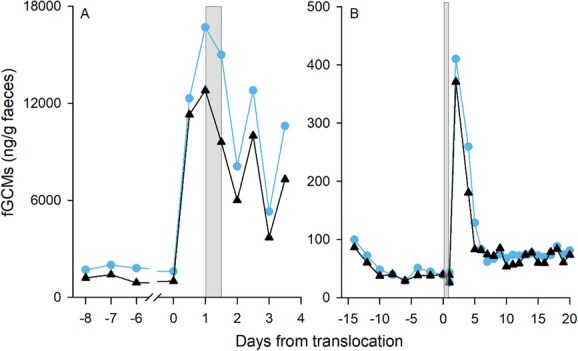
fGCM concentrations in a (**A**) male Alpine chamois, *Rupicapra rupicapra* [comparison data from Anderwald *et al.,* 2021] and (**B**) female Asian elephant, *Elephas maximus* before vs. after translocation (shaded bar) measured using the ISWE010 EIA mini-kit (triangle) and the previously published 11-oxoetiocholanolone EIA (circle) by Möstl *et al.* (2002)

Data from ACTH challenges (Figs 1 and 2) and biological events (Fig. 3) with the newly developed ISWE010 EIA mini-kit were comparable to the 11-oxoetiocholanolone EIA (UVM 72T) developed by Möstl and coworkers (Möstl *et al.,* 2002). Correlations between data analysed on both EIAs were highly correlated, with correlation coefficients ranging from 0.78 to 1.00, with the exception of a male Asian elephant, *Elephas maximus*, where general trends were visually similar, but the peak post-translocation was more pronounced on the UVM 72T assay. Across individuals, overall concentrations did differ, with a greater magnitude response obtained on the ISWE010 EIA mini-kit in 18 out of 21 direct comparisons (Supplementary Material, Table S1). Compared to other, previously published assays that target either parent hormone or alternative metabolite groups, data were generally well correlated, with correlation coefficients >0.7 in 11 of 19 individuals. However, some discrepancies were observed. Among these were the Krefft’s glider described above, and a single brushtail possum with poor correlation compared to three conspecifics; however, only limited samples were available from this individual, so peak fGCM excretion may have been missed.

## Discussion

An 11-oxoetiocholanolone EIA (Möstl *et al.,* 2002) has previously proven well suited to evaluate adrenocortical activity in a variety of species; however, it utilizes a biotinylated label and its application is restricted to highly specialized laboratories, hindering a broader application. Therefore, ISWE, in cooperation with Arbor Assays Inc., set out to create a comparable, and commercially available, 11-oxoetiocholanolone mini-kit to expand the availability of this group-specific approach for measuring glucocorticoid metabolites. This ISWE010 EIA mini-kit was tested with faecal samples from pharmacological and biological challenge events in 25 wildlife species. In 23 species, increased fGCMs, exceeding a 2-fold increase from baseline, were detected in response to pharmacological or biological validation. Further, in 11 of those species, data were compared to the originally developed 11-oxoetiocholanolone (UVM 72 T) EIA and were highly correlated. We included a variety of species in this study, including one bird, two bats, four primates, two *Giraffidae*, six *Bovidae*, four marsupials, one monotreme, one carnivore, two elephants, a hippopotamus and a manatee. The previously described 11-oxoetiocholanolone assay has similarly been validated for a wide range of species, suggesting the versatility of this group-specific antibody for quantifying fGCMs. However, data from one of the marsupials, the Krefft’s glider, were opposite to those expected in comparison to an alternative, validated cortisol assay (Dimovski *et al.,* 2025), highlighting that as hormone metabolism is known to differ between species, biological validation of assay suitability is essential in addition to analytical validation (Palme, 2019). Even for those species where the assay was considered validated, there are some cases where other assays were more sensitive to changes in adrenocortical activity, or where magnitude of change was moderate, warranting further assay comparison and validation. The Ankole cow is an example of this; it is unclear whether this assay is unsuitable for detecting changes in fGCMs in this species, or merely that the event used for biological validation here did not stimulate a significant increase in adrenal activity.

We utilized a range of different validation events, including several biological (inter-zoo translocation, wild capture, social disruption, illness/injury and veterinary intervention) and pharmacological (ACTH) challenges. Thorough validation is a crucial component of any study assessing adrenal activity (Touma and Palme, 2005; Palme, 2019) to ensure that metabolites measured non-invasively are both biologically meaningful and accurately measured by the assay of choice. There can be different factors in the choice of validation, however. For example, ACTH challenges have often been considered the gold-standard for validation as they directly stimulate the production of GCs from the adrenal gland, offering the opportunity to measure circulating or excreted metabolites over a defined timeframe. However, they may require additional permissions and licences and so might not be feasible for all researchers. In contrast, biological validations can take advantage of activities or events that occur naturally or as part of animal management practices. They may not always provide as high a magnitude response as ACTH challenges, but researchers can be assured that significant changes in concentrations reflect biologically relevant responses. Indeed here, peak increases were more than 13-fold post-translocation in a male okapi, *Okapia johnstoni*; 16-fold in a male Florida manatee, *Trichechus manatus latirostri*, following surgical treatment; and 28-fold in a mixed-sex group of ghost bats, *Macroderma gigas*, following a biopsy; indicating that with the appropriate assay, similar magnitude changes can be observed. We also applied a range of extraction techniques as determined by the species and standard laboratory practices at each of our testing facilities; although this may introduce differences in absolute concentrations reported here, clear increases post-challenge were apparent. The decision of sample pre-processing—wet vs. dry extraction, the type (ethanol or methanol) and concentration of solvent, and the presence or absence of a concentration step remains the responsibility of each lab to ensure optimal methodology for the species and hormones of interest (Palme *et al.,* 2013).

Here, we opportunistically compared assay data for a subset of species either to the previously described 11-oxoetiocholanolone assay (Möstl *et al.,* 2002) or to alternative GC assays that had been previously published using the same sample sets. Compared to the Möstl *et al.* (2002) 11-oxoetiocholanolone assay on which this new mini-kit was based, concentrations in some species were slightly higher, others slightly lower, but trends over time and magnitude of response were consistent. This indicates that the new EIA may have slightly higher or lower cross-reactivity with particular fGCMs present in those species, but interpretation of the adrenal response to stimulation is largely similar. It is important to remember that different assays will always yield slightly different results due to their cross-reactivity with particular metabolites, as well as varying environmental conditions between laboratories, so relative changes can be more informative than absolute concentrations (Möstl *et al.,* 2005; Palme, 2019). Used with appropriate care, GCs are useful biomarkers for assessing acute responses to stress, but this is a complex biological process with both normal and pathological actions of interest to researchers investigating animal physiology and its implication on well-being and conservation. As the output of the adrenal gland can potentially differ under different situations (Vera *et al.,* 2011; Koren *et al.,* 2012; Gong *et al.,* 2015), further investigations are required to determine if this assay will also be useful for monitoring changes in normal physiological patterns [e.g. reproductive-related changes (Kersey *et al.,* 2011, Fanson *et al.,* 2014, Edwards *et al.,* 2020)], and longer-term alterations in GCs that can be indicative of chronic stress or adaptation to a changing environment. Research in additional species should include similar validations and consider a comparison of multiple assays to determine the optimal tool(s) for the selected species and research question.

There can be considerable differences regarding metabolism and excretion of GCs across species (Palme *et al.,* 2005), resulting in diverse groups of fGCMs excreted. Parent hormone (e.g. cortisol and corticosterone) antibodies can be advantageous where a high proportion of minimally metabolized hormone is excreted [e.g. barbary macaque, *Macaca sylvanus* (Heistermann *et al.,* 2006, Edwards *et al.,* 2013)] or are often used as multi-species assays due to their cross-reactivity with multiple (often unidentified) metabolites. Conversely, group-specific antibodies are designed for particular metabolite groups, and have often been found to be more sensitive to changes in adrenal activity. It is becoming more common to compare multiple assays during the validation stage, often including both parent and group-specific options [see (Fanson *et al.,* 2017, Hovland *et al.,* 2017, Medger *et al.,* 2018, Puehringer-Sturmayr *et al.,* 2018, Webster *et al.,* 2018, Lavin *et al.,* 2019) for examples of this approach across taxa]. Although we could not compare all samples tested here across multiple assays, we were able to do this opportunistically. All the previously published datasets utilized here had taken that approach, and the relative magnitude and timeframe of increases post-challenge using the 11-oxoetiocholanolone ISWE010 EIA were comparable to those previously reported. With the notable exception of the Krefft’s glider where the cortisol ISWE002 performed significantly better, the ISWE010 assay performed comparably to the previously selected assay. Of the previously unpublished data, although alternative assays were compared and, in some cases, the ISWE010 performed better, this was not done systematically, and so further investigation is warranted in these species.

One important finding during testing of this new mini-kit was that it is important to follow kit instructions for optimal assay performance; repeated freeze-thaws and storage at inappropriate temperatures (specifically −80°C as opposed to the recommended −20°C) can have negative consequences for assay performance. This highlights the need for good quality control measures to ensure assay and therefore data robustness. Overall, the ISWE010 EIA mini-kit performed well on faecal samples from a wide variety of species tested, with comparable responses following pharmacological (ACTH challenges) and biological (stressful events like transportation) validation to that of previously described EIAs. Users must ensure similar thorough validation prior to use to ensure optimal assay choice for each new species of interest.

## Supplementary Material

Web_Material_coaf074

## Data Availability

The data underlying this article are available in the article and in its online supplementary material. Information on underlying assay metrics not found herein will be shared on reasonable request to the corresponding author.
