# Supplementary material for: Development of an 11-oxoetiocholanolone mini-kit for the quantification of faecal glucocorticoid metabolites in various wildlife species
Source: Conserv Physiol. 2025 Oct 24;13(1):coaf074. doi: 10.1093/conphys/coaf074 (PMC12552035; doi:10.1093/conphys/coaf074)
Supplement: Web_Material_coaf074 [file web_material_coaf074.zip › Supplementary Materials_ISWE010.pdf]

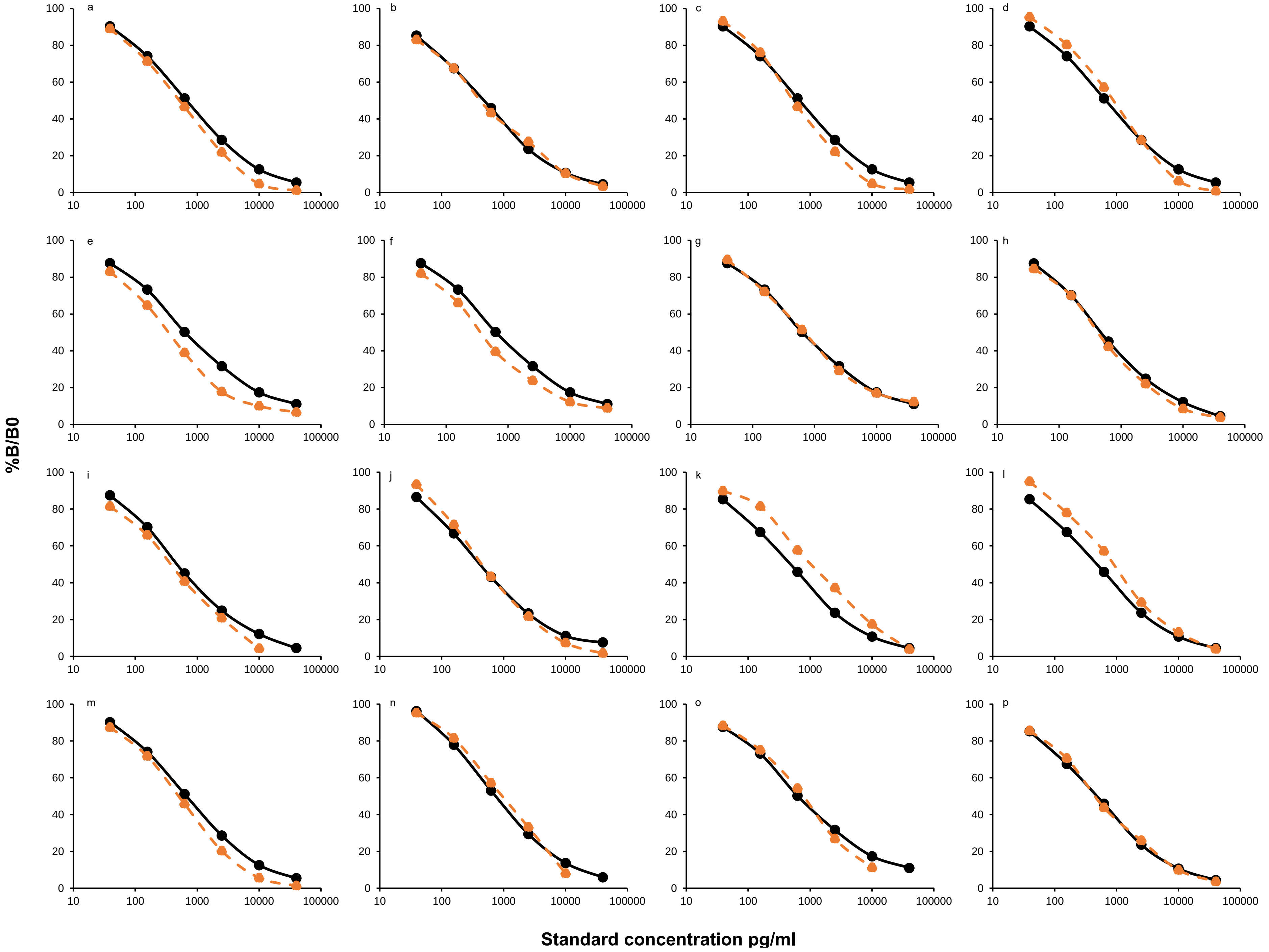
%B/B0
a
b
c
d
e
f
g
h
i
j
k
l
m
n
o
p
0
20
40
60
80
100
10
100
1000
10000
100000
Standard concentration pg/ml

Figure S1: Displacement curves of 4-fold serial dilutions of the 11-oxoetiocholanolone standard (black, solid) and faecal extracts (orange, dashed) for a) African elephant, *Loxodonta africana*; b) ankole cattle, *Bos taurus ankole*; c) Asian elephant, *Elephas maximus* (female); d) Asian elephant, *Elephas maximus* (male); e) brushtail possum, *Trichosurus vulpecula*; f) Florida manatee, *Trichechus manatus latirostri*; g) ghost bat, *Macroderma gigas*; h) giraffe, *Giraffa camelopardalis tippelskirchi*; i) hippopotomus, *Hippopotamus amphibius*; j) Krefft's glider, *Petaurus notatus*; k) mandrill, *Mandrillus sphinx*; l) okapi, *Okapia johnstoni* (female); m) okapi, *Okapia johnstoni* (male); n) roan antelope, *Hippotragus equinus*; o) short-beaked echidna, *Tachyglossus aculeatus*; p) western lowland gorilla, *Gorilla gorilla gorilla*. Curve alignment is centred around the dilution closest to 50% binding, which was then used for analysis of samples.

Table S1: Comparison of 11 species (median baseline and peak concentrations, fold-change **≥ 2.0** considered a response, *<2.0* require further investigation) between the newly developed 11-oxoetiocholanolone (ISWE010) mini-kit EIA and the assay developed by Möstl *et al.* (2002), antibody code UVM 72T. Pearson's correlation (r) was determined for each individual, using all samples tested.

			ISWE010			UVM 72T			
**Species**	**Sex (N)**	**Event**	**Baseline concentration (ng/g)**	**Peak (ng/g)**	**Fold change**	**Baseline concentration (ng/g)**	**Peak (ng/g)**	**Fold change**	**Pearson correlation (r)**
African elephant, *Loxodonta africana*	Female (1)	Translocation	83.8	460.2	**5.5**	63.2	275.5	**4.4**	0.92
	Male (1)	Injury - foot injury and lameness [1]	280.9	710.4	**2.5**	220.1	1083.2	**4.9**	0.84
Alpine chamois, *Rupicapra rupicapra*	Male (1)	Translocation [2]	1088.4	12833.1	**11.8**	1740.7	16744.0	**9.6**	0.98
Asian elephant, *Elephas maximus*	Female (1)	Translocation	38.8	371.1	**9.6**	13.8	128.3	**9.3**	0.97
	Male (1)	Translocation	19.3	66.5	**3.4**	30.0	120.0	**4.0**	0.62
Bengal tiger, *Panthera tigris tigris*	Female (1)	Translocation [3]	1229.3	5249.4	**4.3**	1365.1	5106.7	**3.7**	0.92
	Male (1)		1939.3	4054.8	**2.1**	2071.3	3744.6	*1.8*	0.90
Blue wildebeest, *Connochaetes taurinus*	Female (1)	ACTH challenge [4]	2022.5	5561.2	**2.7**	1376.6	2450.1	*1.8*	0.93
	Male (1)		881.5	10630.2	**12.1**	537.7	6089.1	**11.3**	1.00
Blue-and-yellow macaw, *Ara ararauna*	Female (4)	ACTH challenge [5]	13.8	301.0	**21.8**	50.4	497.4	**9.9**	0.97
			12.8	440.7	**34.4**	47.4	704.0	**14.9**	0.99
			4.2	295.2	**70.3**	16.1	451.6	**28.0**	0.98
			10.8	178.9	**16.6**	35.8	330.8	**9.2**	0.94
Cape buffalo, *Syncerus caffer*	Female (1)	ACTH challenge [6]	1791.9	16017.5	**8.9**	1175.5	14443.6	**12.3**	0.89
	Male (1)		2953.9	34065.6	**11.5**	1866.4	23518.3	**12.6**	0.89
Giraffe, *Giraffa camelopardalis*	Male (1)	ACTH challenge [7]	543.4	13468.2	**24.8**	341.3	3443.0	**10.1**	0.88
Golden langur, *Trachypithecus geei*	Female (1)	ACTH challenge [8]	757.7	2167.2	**2.9**	669.5	1655.5	**2.5**	0.98
Roan antelope, *Hippotragus equinus*	Female (1)	ACTH challenge [9]	1107.7	9665.5	**8.7**	776.2	6134.4	**7.9**	0.97
	Male (1)		722.5	9564.3	**13.2**	607.9	6316.7	**10.4**	1.00
Samango monkey, *Cercopithecus albogularis*	Female (1)	ATCH challenge [10]	1850.2	12618.4	**6.8**	1650.0	6537.5	**4.0**	0.94
	Male (1)		1686.4	7725.3	**4.6**	770.1	3457.8	**4.5**	0.78

[1] Ganswindt *et al.* (2003); [2] Anderwald *et al.* (2021); [3] Jepsen *et al.* (2021); [4] Wolf *et al.* (2021); [5] De Almeida *et al.* (2018); [6] Ganswindt *et al.* (2012); [7] Bashaw *et al.* (2016); [8] Sarmah *et al.* (2017); [9] Kamgang *et al.* (2022); [10] Scheun *et al.* (2020)

Table S2: Comparison of four species (median baseline and peak concentrations, fold-change **≥ 2.0** considered a response, *<2.0* require further investigation) between the newly developed 11-oxoetiocholanolone (ISWE010) mini-kit EIA and previously published assays used for quantifying fGCMs. Pearson's correlation (r) was determined for each individual, using all samples tested.

			ISWE010			Comparison Assay				
**Species**	**Sex (N)**	**Event**	**Baseline concentration (ng/g)**	**Peak (ng/g)**	**Fold change**	**Assay ID**	**Baseline concentration (ng/g)**	**Peak (ng/g)**	**Fold change**	**Pearson correlation (r)**
Brushtail possum, *Trichosurus vulpecula* [1]	Female (1)	ACTH challenge	837.5	3584.6	**4.3**	UVM 72a [5]	101.2	1346.7	**13.3**	0.77
	Female (1)	ACTH challenge	177.1	2661.2	**15.0**		15.5	971.7	**62.7**	0.95
	Female (1)	ACTH challenge	1241.3	4839.3	**3.9**		506.3	1338.5	**2.6**	*0.02*
	Male (1)	ACTH challenge	560.2	1263.0	**2.3**		106.1	481.3	**4.5**	0.80
Gould's wattled bat, *Chalinolobus gouldii* [2]	Female (1)	Wild capture	169.4	1505.4	**8.9**	UVM 69a [6]	109.7	963.4	**8.8**	1.00
	Female (1)	Wild capture	99.0	1208.1	**12.2**		83.7	812.7	**9.7**	0.86
	Female (1)	Wild capture	136.0	229.7	*1.7*		173.7	589.6	**3.4**	0.68
	Male (1)	Wild capture	127.8	344.0	**2.7**		123.2	739.3	**6.0**	0.99
	Male (1)	Wild capture	128.0	571.0	**4.5**		114.8	1244.8	**10.8**	0.69
	Male (1)	Wild capture	118.4	600.0	**5.1**		113.4	1261.6	**11.1**	0.97
Krefft's glider, *Petaurus notatus* [3]	Female (1)	Wild capture	23799.5	4100.5	*0.2*	ISWE002 [7]	47.6	695.2	**14.6**	-0.61
	Female (1)	Wild capture	48553.8	2948.9	*0.1*		55.8	1123.7	**20.1**	-0.51
	Female (1)	Wild capture	6711.9	821.2	*0.1*		28.3	549.8	**19.4**	-0.53
Short-beaked echidna, *Tachyglossus aculeatus* [4]	Female (1)	ACTH challenge	19.6	37.3	*1.9*	CJM006 [8]	20.2	42.5	**2.1**	0.94
	Female (1)	ACTH challenge	76.4	310.3	**4.1**	ISWE007 [9]	16.0	103.0	**6.4**	0.88
	Male (1)	ACTH challenge	37.1	98.6	**2.7**	UVM 72a [5]	8.6	43.6	**5.1**	0.49
	Male (1)	ACTH challenge	9.0	19.7	**2.2**	ISWE007	4.2	24.7	**5.8**	0.89
	Female (1)	Housing move	19.4	139.2	**7.2**	UVM 72a [5]	2.7	21.7	**8.0**	0.90
	Male (1)	Housing move	8.5	35.2	**4.1**	CJM006	12.1	86.4	**7.1**	0.49

[1] Cope *et al.* (2022); [2] Sandy *et al.* (2024); [3] Dimovski *et al.* (2025); [4] Russell *et al.* (2022); [5] Palme *et al.* (1997); [6] Frigerio *et al.* (2004); [7] https://www.arborassays.com/product/cortisol-iswe-mini-kit/; [8] Watson *et al.* (2013); [9] https://www.arborassays.com/product/corticosterone-iswe-mini-kit/. Note that for Short-beaked echidna, three assays were used in the previous publication, so these were used for comparison here.

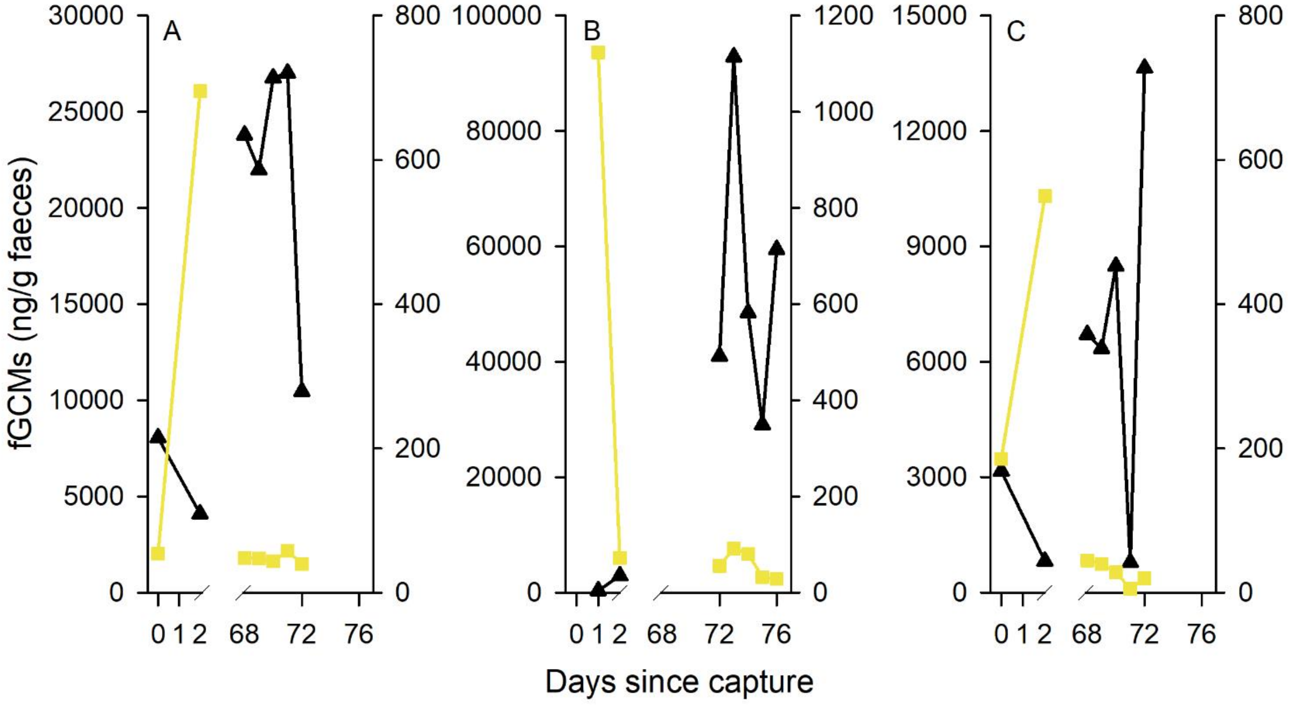

Figure S2: fGCMs in three female Krefft's gliders in response to wild capture and compared on two enzyme immunoassays, 11-oxoetiocholanolone ISWE010 mini-kit (▲; black, closed triangle) and cortisol ISWE002 mini-kit (■; yellow, closed square). Animals were collected from nest boxes and transferred to large outdoor enclosures; the first two samples were collected within 2 days of capture, and the subsequent five samples were collected after 2 months in captivity. Comparison data from Dimovski *et al.* (2025).

References:

Anderwald P, Campell Andri S, Palme R (2021) Reflections of ecological differences? Stress responses of sympatric Alpine chamois and red deer to weather, forage quality, and human disturbance. *Ecology and Evolution* 11: 15740-15753

Bashaw MJ, Sicks F, Palme R, Schwarzenberger F, Tordiffe AS, Ganswindt A (2016) Non-invasive assessment of adrenocortical activity as a measure of stress in giraffe (*Giraffa camelopardalis*). *BMC Veterinary Research* 12: 235

Cope HR, Keeley T, Keong J, Smith D, Silva FR, McArthur C, Webster KN, Mella VS, Herbert CA (2022) Validation of an enzyme immunoassay to measure faecal glucocorticoid metabolites in common brushtail possums (*Trichosurus vulpecula*) to evaluate responses to rehabilitation. *Animals* 12: 1627

De Almeida AC, Palme R, Moreira N (2018) How environmental enrichment affects behavioral and glucocorticoid responses in captive blue-and-yellow macaws (*Ara ararauna*). *Applied Animal Behaviour Science* 201: 125-135

Dimovski AM, Fanson KV, Edwards AM, Robert KA (2025) Short- and long-wavelength lights disrupt endocrine signalling but not immune function in a nocturnal marsupial. *Conservation Physiology* 13:

Frigerio D, Dittami J, Mostl E, Kotrschal K (2004) Excreted corticosterone metabolites co-vary with ambient temperature and air pressure in male Greylag geese (Anser anser). *General and Comparative Endocrinology* 137:

Ganswindt A, Palme R, Heistermann M, Borragan S, Hodges JK (2003) Non-invasive assessment of adrenocortical function in the male African elephant (*Loxodonta africana*) and its relation to musth. *General and Comparative Endocrinology* 134: 156-166

Ganswindt A, Tordiffe A, Stam E, Howitt M, Jori F (2012) Determining adrenocortical activity as a measure of stress in African buffalo (*Syncerus caffer*) based on faecal analysis. *African Zoology* 47: 261-269

Jepsen EM, Scheun J, Dehnhard M, Kumar V, Umapathy G, Ganswindt A (2021) Non-invasive monitoring of glucocorticoid metabolite concentrations in native Indian, as well as captive and re-wilded tigers in South Africa. *General and Comparative Endocrinology* 308: 113783

Kamgang VW, Bennett NC, van der Goot AC, Majelantle TL, Ganswindt A (2022) Patterns of faecal glucocorticoid metabolite levels in captive roan antelope (*Hippotragus equinus*) in relation to reproductive status and season. *General and Comparative Endocrinology* 325: 114052

Möstl E, Maggs JL, Schrotter G, Besenfelder U, Palme R (2002) Measurement of cortisol metabolites in faeces of ruminants. *Veterinary Research Communications* 26: 127-139

Palme R, Möstl E (1997) Measurement of cortisol metabolites in faeces of sheep as a parameter of cortisol concentration in blood. *Zeitschrift Fur Saugetierkunde-International Journal of Mammalian Biology* 62:

Russell FA, Johnston SD, Hill A, Roser A, Meer H, Fenelon JC, Renfree MB, Keeley T (2022) Validation of a non-invasive assessment technique for quantifying faecal glucocorticoid metabolite concentrations in the short-beaked echidna (*Tachyglossus aculeatus*). *General and Comparative Endocrinology* 327: 114092

Sandy LK, Fanson KV, Griffiths SR, Robert KA, Palme R, Dimovski AM (2024) Non-invasive monitoring of adrenocortical activity in the Gould's wattled bat (*Chalinolobus gouldii*). *General and Comparative Endocrinology* 359: 114619

Sarmah J, Hazarika CR, Berkeley EV, Ganswindt SB, Ganswindt A (2017) Non-invasive assessment of adrenocortical function as a measure of stress in the endangered golden langur. *Zoo Biology* 36: 278-283

Scheun J, Tordiffe AS, Wimberger K, Ganswindt A (2020) Validating a non-invasive technique for monitoring physiological stress in the samango monkey. *Onderstepoort Journal of Veterinary Research* 87: 1-8

Watson R, Munro C, Edwards KL, Norton V, Brown JL, Walker SL (2013) Development of a versatile enzyme immunoassay for non-invasive assessment of glucocorticoid metabolites in a diversity of taxonomic species. *General and Comparative Endocrinology* 186: 16-24

Wolf TE, De Haast AR, Meyer LC, Gerber D, Ganswindt A (2021) Measuring faecal glucocorticoid metabolite concentrations as an indicator of stress in blue wildebeest (*Connochaetes taurinus*). *African Journal of Wildlife Research* 51: 90-99
